# Spinal Anesthesia After Thoracolumbar Fixation for Scoliosis: A Case Report Emphasizing the Role of Chest X-ray Reassessment

**DOI:** 10.7759/cureus.101132

**Published:** 2026-01-08

**Authors:** Hanin Alhodathi, Omar Al Misnid, Leen Alhodathi

**Affiliations:** 1 College of Medicine, Qassim University Medical City, Buraydah, SAU; 2 Department of Emergency, Critical Care, and Anesthesia, College of Medicine, Qassim University, Buraydah, SAU

**Keywords:** case report, prior thoracolumbar fixation, scoliosis, spinal anesthesia, taylor’s approach, x-ray guided

## Abstract

Spinal anesthesia (SA) is widely favored for lower limb orthopedic surgeries due to its rapid onset, effectiveness, and lower risk compared to general anesthesia (GA), particularly in patients with comorbidities. Nevertheless, prior spinal surgery presents significant challenges to SA because of deformed anatomy, scarring, and altered surface landmarks.

In this article, we report successful landmark-based SA using radiographic reassessment, without ultrasound, in an elderly patient with multiple comorbidities and previous thoracolumbar fixation for scoliosis who was scheduled for elective total knee arthroplasty.

## Introduction

Spinal anesthesia (SA) is a frequently utilized regional anesthetic technique because of its efficiency, rapid onset, and good safety profile [1,2]. It is commonly preferred because it offers sufficient surgical anesthesia while lowering the risk associated with general anesthesia (GA), especially in patients with comorbidities [3-5]. However, it remains technically difficult to provide SA to patients who have anatomical changes resulting from previous spinal procedures. Due to surgical scarring, fibrosis, disrupted interspinous gaps, and the presence of metallic hardware, which may make neuraxial access more difficult, prior spinal instrumentation can drastically alter normal vertebral structure [6].

Despite these difficulties, GA carries certain risks, particularly for patients who may have systemic or respiratory illnesses. Therefore, SA may still offer significant benefits in appropriately selected patients [7]. Current published research describes varying success rates of neuraxial anesthesia in patients who have undergone spine surgery. It frequently reports the need for multiple attempts or imaging guidance, most commonly fluoroscopy or ultrasound, to aid needle insertion [8].

This case highlights the role of radiographic reassessment in enabling successful landmark-based SA after an initial failed attempt using the Taylor approach in a patient with extensive thoracolumbar instrumentation.

## Case presentation

A 65-year-old female with a body weight of 90 kg and a height of 156 cm, corresponding to a body mass index consistent with Class II obesity, presented for elective total knee arthroplasty under SA. During the preoperative evaluation, her medical history was significant for longstanding hypertension, hypothyroidism, atrial fibrillation, anxiety, and depression.

Her medical conditions were well controlled with telmisartan 80 mg and bisoprolol (Concor) 2.5 mg, taken daily for hypertension. She was also taking levothyroxine 50 mcg and sertraline 100 mg daily for hypothyroidism and psychiatric disorders, respectively. Regarding atrial fibrillation, she was taking apixaban 10 mg daily. Moreover, she was newly diagnosed with asthma, for which she was prescribed tiotropium bromide 18 mcg, montelukast 10 mg, and a salmeterol/fluticasone 25/250 mcg evohaler.

Regarding the patient’s surgical history, she had undergone two major spinal surgeries for scoliosis correction and old vertebral fractures at multiple levels, including dorsolumbar laminectomy and posterior spinal fusion with internal fixation using metallic rods and pedicle screws spanning multiple thoracolumbar levels. These procedures were performed under GA in April 2021, and the patient was admitted to the ICU for two days due to postoperative complications. Thereafter, the patient carried a heavy object, which resulted in a vertebral fracture, and she subsequently underwent another vertebral fixation surgery in December 2021.

On preanesthetic evaluation in the clinic, the patient was classified as American Society of Anesthesiologists (ASA) Physical Status III. Airway assessment demonstrated a Mallampati Class III, suggesting potentially moderate difficulty. Cardiovascular examination revealed normal heart sounds with no murmurs and no signs of heart failure. Respiratory examination demonstrated clear breath sounds bilaterally, with normal respiratory effort and oxygen saturation on room air. Preoperative vital signs were stable, with a blood pressure of 143/95 mmHg, heart rate of 102 bpm, and oxygen saturation of 98% on room air. Asthma was considered clinically stable under inhaled bronchodilator and corticosteroid therapy, which was continued perioperatively. Laboratory investigations were unremarkable.

On the day of surgery, antihypertensive, thyroid replacement, and psychiatric medications were continued for perioperative optimization. In compliance with current perioperative anticoagulation guidelines, apixaban was withheld 24 hours before surgery, in accordance with the American Society of Regional Anesthesia (ASRA) guidelines for neuraxial anesthesia, taking into account the patient’s preserved renal function and absence of additional bleeding risk factors [9].

Neuraxial anesthesia was anticipated to present a technical challenge due to the patient’s extensive history of thoracolumbar spinal operations with posterior fusion and instrumentation. In order to reduce the cardiovascular risks associated with GA and to prepare for the possibility of failure owing to altered spinal anatomy, an anesthetic plan favoring SA was developed. Vasoactive medications and emergency airway equipment were made readily available, and routine monitoring was planned. The patient was counseled regarding the anticipated challenges, the risk of block failure, and the possibility of conversion to GA, and informed consent was obtained.

Upon arrival in the operating room, standard monitoring was applied, including pulse oximetry, non-invasive blood pressure monitoring (NIBP), and continuous electrocardiography (ECG). A 20-gauge intravenous cannula was inserted in the right hand. During back examination, a well-healed linear midline surgical scar was noted, extending from the mid-thoracic region to the sacrum. On palpation, the spinous processes and intervertebral spaces were poorly palpable, making identification of anatomical landmarks difficult. Preoperative chest radiography was reviewed to better understand the anatomical abnormalities. The patient was then positioned in the sitting position, with the back flexed and the head bent forward. Strict aseptic technique was applied in the thoracolumbar region. A Taylor approach was chosen to facilitate access to the subarachnoid space, as the midline approach was expected to be challenging.

**Figure 1:**
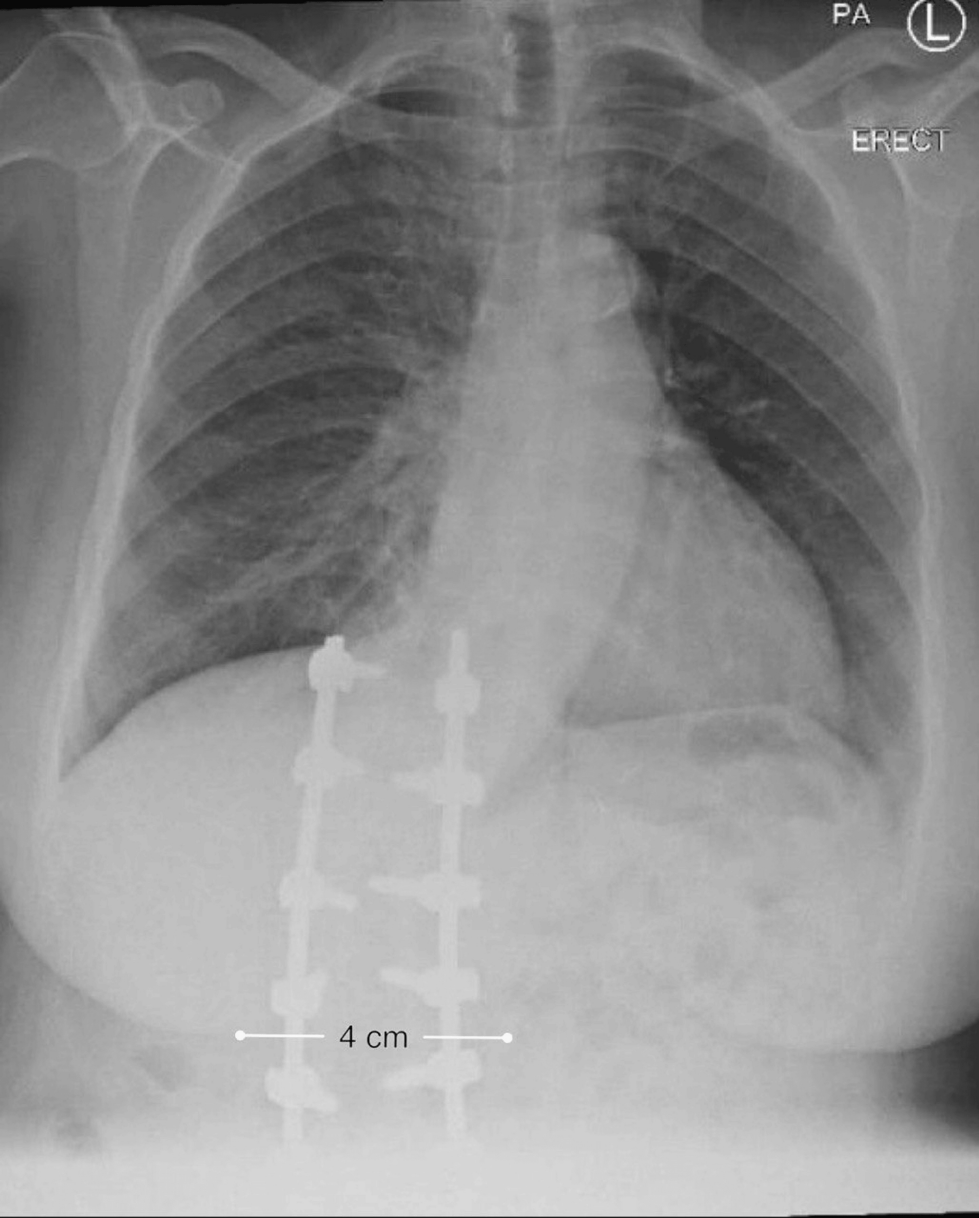
Preoperative posteroanterior chest X-ray showing thoracolumbar spinal fixation with vertebral fusion and lateral deviation of the spinal column.

Given the anticipated difficulty due to abnormal anatomical landmarks, SA was initially attempted using a right paramedian Taylor approach. After local infiltration of the skin with 1% lidocaine, the needle entry point was estimated to be approximately 3.5-4 cm lateral to the midline at the L5-S1 interspace. A 27-gauge Quincke spinal needle was then advanced. However, this initial attempt was unsuccessful. Therefore, ultrasound was prepared, and preoperative radiographic imaging, specifically a chest X-ray, was reviewed again after the first failed attempt, to better understand the patient’s altered spinal anatomy and inform the needle trajectory (Figure 1). Given the two-dimensional nature of chest radiography and its limited relevance to lumbar anatomy, this imaging provided only indirect guidance. In such technically challenging cases, dedicated lumbar imaging would typically be preferred for precise level selection and needle trajectory. Based on the chest X-ray findings, which demonstrated fusion of the lumbar and lower thoracic vertebrae, with an approximate 4-5 cm deviation of the vertebral column toward the right side, the needle trajectory was adjusted to a more vertical orientation at the same insertion point.

Following this adjustment, Dr. Omar Al Misnid achieved successful entry into the intrathecal space, as confirmed by the free flow of clear cerebrospinal fluid on the first subsequent attempt, without the use of ultrasound due to operator experience. A total of 2.8 mL of 0.5% hyperbaric bupivacaine was administered, chosen for its more stable intraoperative hemodynamic profile and longer sensory block compared with isobaric bupivacaine. The patient was then repositioned to the supine position. Prior to the surgical incision, adequate sensory and motor block was attained, and no acute complications were noted. Supplementary oxygen was administered at 2 L/min, and the patient maintained hemodynamic stability throughout the 100-minute surgical procedure.

An appropriate sensory block up to the T10 level was obtained after successful intrathecal injection, which was sufficient for the intended total knee replacement. The patient maintained hemodynamic stability throughout the procedure, with heart rate and blood pressure remaining within acceptable limits. Ringer’s lactate was administered in a total volume of approximately 1500 mL. There were no episodes of severe hypotension, bradycardia, desaturation, or arrhythmia, and no vasopressor therapy was required. Conversion to GA was not necessary to complete the procedure under SA.

The patient was transferred to the post-anesthesia care unit (PACU), where she was stable, alert, and oriented. No nausea, vomiting, or respiratory complications were noted, and pain control was adequate. Neurological evaluation revealed no new deficits, with full regression of both sensory and motor block. Following an unremarkable postoperative course, the patient was discharged from the recovery unit in accordance with institutional practice.

## Discussion

SA is preferred and widely used by anesthesiologists for total knee arthroplasty, especially in geriatric patients, and is found to have many advantages and to be equally effective as GA [10]. Moreover, it has a low risk of respiratory complications, thromboembolic events, and renal failure [11]. These favorable outcomes are clinically beneficial for patients with comorbidities and contribute to a shortened postoperative hospital stay [12].

The Taylor approach is considered an excellent alternative technique, with a high success rate in geriatric patients and in those with abnormal spinal anatomy, such as severe scoliosis and a prior history of spine surgery [13-15]. We decided to attempt the Taylor approach, which is performed at the L5-S1 interspace, the largest interspace of the vertebral column. This approach is applied by palpating the posterior superior iliac spine (PSIS) to guide needle insertion in a cephalomedial direction, approximately 1 cm medial and 1 cm caudal to the prominence of the PSIS. In some patients, palpation may be difficult due to abnormal surface landmarks, morbid obesity, or positional limitations [16]. As in our case, palpation was difficult due to thoracolumbar fixation and right-sided tilting of the lumbar vertebrae, which led to an unsuccessful attempt.

Following this attempt, preoperative chest X-ray imaging revealed important information regarding spinal alignment, vertebral fusion, and lateral deviation, which guided procedural planning and needle trajectory. By combining radiographic findings with meticulous clinical examination and procedural reevaluation, successful SA was accomplished without the need for ultrasound guidance, due to operator experience. Although ultrasound guidance has been increasingly recommended in the literature to help identify intervertebral spaces and optimize needle trajectory [17], severe anatomical distortion or operator dependence may limit the utility of ultrasonography.

The present case demonstrated that SA can be successfully administered in a patient with prior thoracolumbar fixation, when careful reassessment and technique modification are undertaken. Several published reports have shown that neuraxial anesthesia may be feasible in patients with significant spinal deformities, when guided by preoperative assessment. For example, Chaudhary et al. [18] described successful SA during cesarean section in a patient with thoracolumbar gibbus deformity, based on preoperative MRI evaluation, and Kirby et al. [19] reported successful SA in elderly patients with severe lumbar scoliosis and multiple comorbidities, using preoperative lumbar radiographs. Additionally, Kim et al. [20] demonstrated high success rates and no serious neurological complications in patients undergoing total knee arthroplasty after prior lumbar spine surgery. However, these studies largely involved patients without spinal instrumentation, representing a lower degree of anatomical distortion than that encountered in the present case.

Other reports have highlighted the limitations of conventional spinal techniques in more rigid or complex spinal conditions. Channabasappa et al. [8] described the failure of traditional SA and the need for fluoroscopy-guided epidural techniques in patients with extreme spinal rigidity, such as spinal ankylosis. Similarly, Jindal et al. [16] reported successful SA using Taylor’s approach in patients with ankylosing spondylitis, following failure of standard techniques. While these studies support the feasibility of alternative neuraxial strategies in difficult spines, they do not directly address the added challenges posed by prior thoracolumbar fixation with metallic instrumentation.

In the present case, SA was not achieved on the initial attempt, and modification of the technique occurred only after recognition of procedural difficulty. Review of available radiographic imaging assisted in understanding overall spinal alignment and lateral deviation; however, its role was adjunctive rather than definitive, and the limitations of chest radiography in defining lumbar neuraxial anatomy are acknowledged. Operator experience contributed primarily to early recognition of failed needle placement, avoidance of repeated traumatic attempts, and appropriate procedural reassessment, rather than guaranteeing success.

Accordingly, this case should not be interpreted as evidence for routine landmark-based SA in patients with prior spinal instrumentation. Instead, it illustrates that neuraxial anesthesia may remain feasible in selected cases when undertaken with cautious progression, readiness to reassess technique, and preparedness to convert to alternative anesthetic strategies, if required.

This case underscores several considerations relevant to anesthetic decision-making in complex spinal anatomy. The use of SA should not be automatically precluded by prior spinal fixation and altered surface landmarks, particularly when careful preprocedural assessment is performed. An initial unsuccessful attempt does not necessarily indicate failure, and reassessment, guided by available imaging and clinical examination, may allow successful neuraxial access. These observations are limited to a single case and should not be interpreted as supporting routine neuraxial anesthesia in patients with prior spinal instrumentation.

The limitations of this case report include its single-case nature, which restricts generalizability to all patients with prior spinal instrumentation. Only conventional chest X-ray imaging was used for radiological assessment, and no advanced imaging modalities were utilized; therefore, future studies should evaluate the comparative value of different imaging techniques in managing complex spinal anatomy. Long-term neurological follow-up was not available, and radiation exposure considerations, even if minimal, should be acknowledged. Additionally, the applicability of the findings is influenced by operator experience and patient selection, which may limit broader interpretation of the results. Future studies should include larger case series or observational studies to better define the role of landmark-based SA in this patient population.

## Conclusions

This case illustrates that SA may be achievable in selected patients with previous thoracolumbar fixation, despite altered anatomy. Review of radiographic imaging served as an adjunct to understanding overall spinal alignment, rather than a definitive guide to neuraxial anatomy. This case emphasizes the importance of individualized decision-making, cautious technique modification following recognition of technical difficulty, and readiness to employ alternative anesthetic strategies, with imaging review considered supportive rather than a substitute for ultrasound guidance.
